# Alteration of Striatal Dopaminergic Neurotransmission in a Mouse Model of DYT11 Myoclonus-Dystonia

**DOI:** 10.1371/journal.pone.0033669

**Published:** 2012-03-16

**Authors:** Lin Zhang, Fumiaki Yokoi, Dee S. Parsons, David G. Standaert, Yuqing Li

**Affiliations:** 1 Department of Neurology, School of Medicine, University of Florida, Gainesville, Florida, United States of America; 2 Center for Neurodegeneration and Experimental Therapeutics, Department of Neurology, School of Medicine, University of Alabama at Birmingham, Birmingham, Alabama, United States of America; Chiba University Center for Forensic Mental Health, Japan

## Abstract

**Background:**

DYT11 myoclonus-dystonia (M-D) syndrome is a neurological movement disorder characterized by myoclonic jerks and dystonic postures or movement that can be alleviated by alcohol. It is caused by mutations in *SGCE* encoding ε-sarcoglycan (ε-SG); the mouse homolog of this gene is *Sgce*. Paternally-inherited *Sgce* heterozygous knockout (*Sgce* KO) mice exhibit myoclonus, motor impairment and anxiety- and depression-like behaviors, modeling several clinical symptoms observed in DYT11 M-D patients. The behavioral deficits are accompanied by abnormally high levels of dopamine and its metabolites in the striatum of *Sgce* KO mice. Neuroimaging studies of DYT11 M-D patients show reduced dopamine D2 receptor (D2R) availability, although the possibility of increased endogenous dopamine, and consequently, competitive D2R occupancy cannot be ruled out.

**Methodology/Principal Findings:**

The protein levels of striatal D2R, dopamine transporter (DAT), and dopamine D1 receptor (D1R) in *Sgce* KO mice were analyzed by Western blot. The striatal dopamine release after amphetamine injection in *Sgce* KO mice were analyzed by microdialysis *in vivo*. The striatal D2R was significantly decreased in *Sgce* KO mice without altering DAT and D1R. *Sgce* KO mice also exhibited a significant increase of dopamine release after amphetamine injection in comparison to wild-type (WT) littermates.

**Conclusion/Significance:**

The results suggest ε-SG may have a role in the regulation of D2R expression. The loss of ε-SG results in decreased striatal D2R, and subsequently leads to increased discharge of dopamine which could contribute to the behavioral impairment observed in DYT11 dystonia patients and in *Sgce* KO mice. The results suggest that reduction of striatal D2R and enhanced striatal dopamine release may contribute to the pathophysiology of DYT11 M-D patients.

## Introduction

Dystonia is a neurological disorder characterized by involuntary contractions of both agonist and antagonist muscles of affected body regions that cause twisting and abnormal movements or postures [1]. M-D is a childhood onset movement disorder, clinically characterized by the presence of ‘lighting-like’ myoclonic jerks and dystonic postures or movements that can be alleviated by alcohol [2]. DYT11 M-D patients often show psychiatric abnormalities including alcohol abuse, depression, anxiety, panic attacks, and obsessive-compulsive disorder [3], [4], [5]. DYT11 M-D is caused by mutations in *SGCE* (*Sgce* in the mouse) on chromosome 7q21 coding for ε-SG [3]. It is inherited in an autosomal dominant manner with *SGCE* maternally imprinted probably by promoter methylation [6], [7]. Exclusive paternal expression of ε-SG in the brain is confirmed in both mice [8], [9] and humans [10]. Both nonsense and missense mutations have been found in DYT11 M-D patients. The missense mutations usually result in a shift of translational reading frame and introduce premature termination codon [3], [11]. Several *SGCE* missense mutations in extracellular domain of ε-SG impair membrane trafficking of the mutant proteins in cultured cells [12]. Taken together, these results suggest that the loss of function of ε-SG causes DYT11 M-D.

The sarcoglycans are a family of plasma membrane proteins, which consists of six different isoforms, α, β, γ, δ, ε and ζ [13]. The α, β, γ, and δ-sarcoglycans form a heterotetrameric complex that associates with dystroglycan at the plasma membrane in muscle. Mutations in α, β, γ, and δ-sarcoglycans lead to muscle membrane instability and result in autosomal recessive limb-girdle muscular dystrophies (LGMD). ε-SG is widely expressed in many tissues including brain [3], [14]. Fluorescence *in situ* hybridization (FISH) and immunohistochemistry studies have revealed that ε-sarcoglycan mRNA is expressed at high level in cholinergic neurons of dorsal raphe nucleus and in dopaminergic neurons of the substantia nigra pars reticulata, pars compacta, and ventral tegmental area [15]. The function of ε-SG is largely unknown. Subcellular fractionation of the mouse brain homogenate have revealed ε-SG is relatively enriched in post-and pre-synaptic membrane fractions, suggests its possible role in the synaptic transmission of the central nervous system [16]. This is supported by recent brain-specific knockout of *Sgce* in mice. Specific knockout of *Sgce* in striatum or cerebellar Purkinje cells results in deficits of motor learning, coordination and balance [17], [18].

Although the pathophysiological nature of dystonia is largely elusive, basal ganglia, especially striatal dopaminergic system is thought to play a major role. DYT5 dystonia is caused by mutations in either GTP cyclohydrolase gene [19] or tyrosine hydroxylase [20], [21]. Mutations in both genes have direct impact on dopamine synthesis. Clinical neuroimaging studies in dystonia patients, including DYT1 dystonia, idiopathic cervical dystonia, and nocturnal myoclonus have showed reduced *in vivo* striatal D2R binding [22], [23], [24]. Furthermore, a recent neuroimaging study has revealed reduced striatal D2R binding in DYT11 M-D patients [25]. In addition, a missense mutation (Val154Ile) in a highly conserved region of the D2R was found in one M-D family [2]. D2R-mediated dopaminergic neurotransmission is known to have a key role in the control of movement [26], in reward and reinforcement mechanisms [27], and in psychiatric disorders [28]. Pharmacological agents blocking D2R can result in a dystonic phenotype [29].

We previously reported generation of *Sgce* KO mice by using *Cre*-*loxP* system to flank exon 4 of *Sgce* and demonstrated that expression of ε-SG is fully abolished in paternally inherited *Sgce* heterozygous KO mice [9]. The *Sgce* KO mice exhibit myoclonus, motor impairments, anxiety- and depression-like behaviors [30], which resemble several clinical symptoms observed in DYT11 M-D patients [2]. Moreover, these are accompanied by significantly high levels of dopamine and its metabolites in the striatum [30], implicating abnormal striatal dopaminergic function in *Sgce* KO mice. In this study, we aim to determine the nature of the dopaminergic dysfunction and found significantly reduced level of striatal D2R with normal level of D1R and DAT in *Sgce* KO mice. Furthermore, we found increased discharge of striatal dopamine in the mutant mice after amphetamine injection. The results suggest that striatal dopaminergic dysfunction contributes to the pathophysiology of DYT11 M-D.

## Results

### Increased striatal dopamine release in *Sgce* KO mice

**Figure 1:**
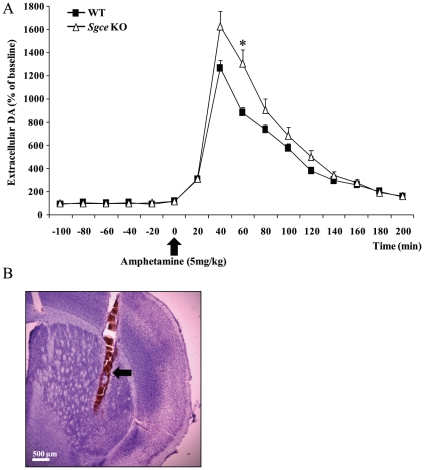
Extracellular dopamine levels in the mouse striatum after amphetamine administration. (A) Amphetamine (5 mg/kg, s.c.) was administrated to conscious mice. Extracellular levels of dopamine in the striatum were measured by *in vivo* microdialysis. The basal extracellular dopamine levels were 4.013±0.267 pg/20 µl (mean ± SEM of 6 *Sgce* KO mice) and 4.297±0.412 pg/20 µl (mean ± SEM of 8 WT littermates). The data are the mean ± SEM of 6 or 8 mice (*p<0.05). (B) A representative coronal section of the striatum of probe implanted mouse. The black arrow indicates the location of the probe. Scale bar represents 500 µm.

Previous study has shown increased dopamine and its metabolites in the striatum of *Sgce* KO mice accompanied with behavioral deficits similar to DYT11 M-D patients [30]. To examine whether the striatal dopaminergic transmission is changed in *Sgce* KO mice, we used microdialysis to monitor extracellular dopamine levels after amphetamine injection in conscious, freely moving mice as previously described [31]. Amphetamine, a potent psychostimulant, enhances the release of dopamine from pre-synaptic dopaminergic terminals [32]. A single subcutaneous (s.c.) administration of amphetamine (5 mg/kg) induced a remarkable increase of striatal extracellular dopamine level in *Sgce* KO mice (1,620±45%, n = 6) in comparison to their WT littermates (1,260±118%, n = 8, *p*<0.05) 60 minutes after injection (Fig. 1A). Data from each animal were normalized to the corresponding pre-treatment baseline and expressed as a percent of base line of extracellular dopamine level. Repeated ANOVA analysis revealed a significant difference of amphetamine pre-treatment and post-treatment [*F* (1, 12) = 3.90, *p*<0.05]. No significant difference in the basal extracellular dopamine levels was found. Probe locations were verified in all mice at the end of the microdialysis experiment (Fig. 1B).

### Decreased striatal D2R without altering D1R and DAT

**Figure 2:**
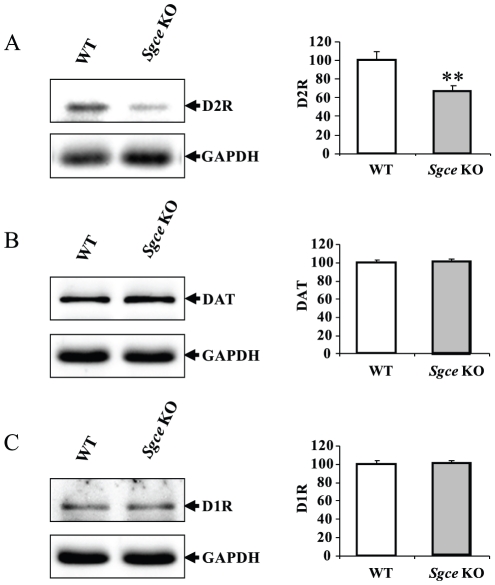
Western blot analysis of striatal D1R, D2R and DAT. Western blot analysis by using the striatal protein extracts from paternally inherited *Sgce* KO mice and their WT littermates. The representative bands D2R (Fig. 2A), D1R (Fig. 2C), DAT (Fig. 2B), and GAPDH are shown in the left side, and the quantified results are shown in the right side. The vertical bars represent means ± SEM of 3 or 4 mice (**p<0.01).

Neuroimaging studies have revealed decreased *in vivo* striatal D2R availability in clinical DYT11 M-D patients [25]. In addition, a recent study has reported dopaminergic neuron-specific D2R knockout results in elevated dopamine synthesis and release, hyperactivity and supersensitivity to cocaine [33]. To examine whether loss of ε-SG alters the expression of D2R in the striatum, we analyzed expression level of D2R in *Sgce* KO and control littermates by Western blot. The D1R, D2R, and DAT levels were standardized with glyceraldehyde-3-phosphate dehydrogenase (GAPDH) as a loading control. Striatal D2R was significantly reduced in *Sgce* KO mice in comparison to control littermates (*p*<0.01; Fig. 2A), while striatal D1R and DAT remained unchanged (Fig. 2B, C).

## Discussion

Striatal dopamine and their metabolites are increased in *Sgce* KO mice [30]. To determine the nature of this dopaminergic dysfunction, we used microdialysis to examine the striatal dopamine release in *Sgce* KO mice. We found significantly increased extracellular dopamine after administration of amphetamine in *Sgce* KO mouse compared to their WT littermates. Next, to determine if this abnormal release of dopamine in the striatum observed was associated with dysfunction of dopamine receptors or transporter, we used Western blot technique to assess the expression levels of D2R, DAT, and D1R in the striatum and found that D2R was significant decreased without altering D1R and DAT. These results suggest ε-SG may have a role in the regulation of D2R expression. The loss of ε-SG results in decreased striatal D2R, and subsequently leads to increased discharge of dopamine which could contribute to the behavioral impairment observed in *Sgce* KO mice and DYT11 M-D patients.

Despite of significantly increased striatal dopamine turnover, the level of striatal dopamine is significantly higher in the *Sgce* KO mice, which indicated a lack of autoreceptor function [30]. Pre-synaptic D2R is present in the soma, dendrites and synaptic terminals of dopaminergic neurons, serving as autoreceptor and providing negative feedback regulation of firing rate [34], [35], synthesis and release of dopamine in the terminals [33], [36]. Amphetamine is well known to elevate extracellular dopamine levels by reversing DAT and inhibiting dopamine re-uptake through DAT, which results in significantly inducing stimulation-independent non-vesicular dopamine efflux. Dramatic increase of dopamine activates the pre-synaptic D2 receptor and inhibits the activity of dopamine neurons and subsequently inhibits stimulation-dependent exocytosis of dopamine in the terminals [37]. This inhibition appears to be defective in *Sgce* KO mice (Fig. 1A, 2A). The D2R results we obtained from Western blot include both post-synaptic D2R on the medium spiny neurons and pre-synaptic D2R on the dopaminergic terminals from substantia nigra. Since majority of striatal D2R are located on the post-synaptic medium spiny neurons, the reduction of striatal D2R may just reflect those in the post-synaptic neurons (Fig. 2A). However, decreased striatal post-synaptic D2R alone will not affect dopamine release from terminals of dopaminergic neurons after amphetamine challenging (Fig. 1A) and result in significantly increased striatal tissue dopamine observed in *Sgce* KO mice [30]. Furthermore, conditional knockout mice lacking D2 autoreceptor are hyperactive in open field test and show enhanced dopamine release in dorsal striatum and elevated tyrosine hydroxylase activity [33]. Although conditional D2 autoreceptor KO mice exhibit normal motor coordination and no signs of anxiety-like behavior, which differ from the behavioral abnormalities observed in *Sgce* KO mice [30], the differences may be attributed to intact post-synaptic D2R in such D2R conditional KO mice. Alternatively, additional pathways other than D2R may have been disrupted in *Sgce* KO mice that lead to motor deficits and anxiety-like behaviors. Taken together, it is likely that both pre- and post-synaptic D2R are reduced in the *Sgce* KO mice.

It is not known how loss of ε-SG led to D2R reduction in the *Sgce* KO mice. Although the functions of other sarcoglycans are well documented, the functions of ε- and ζ-sarcoglycans, which express widely in central nervous system, are less clear. Sarcoglycan family is well known for its major role in stabilizing membrane through forming complex with dystroglycan and dystrophin during the muscle contraction. Mutations in α, β, γ, and δ-sarcoglycan result in autosomal recessive limb-girdle muscular dystrophies (LGMD) [13]. mRNA for ε-SG is expressed at high level in dopaminergic neurons [15], suggesting that it may have an important role in stabilizing the membrane of dopaminergic neurons. Indeed, our results show that elimination of ε-SG resulted in the reduction of D2R in the striatum (Fig. 2A). We previously reported brain-specific alternatively spliced mRNA variants of ε-SG in mice. These two variants possess unique C-terminal sequences which are similar to PDZ-binding motifs [9]. Many G-protein coupled receptors contain a PDZ binding motif at their C-terminals enabling other PDZ proteins to associate and scaffold multi-protein complexes that can modulate receptor properties such as trafficking, signaling, receptor stability and cell distribution [38]. Whether ε-SG acts in a similar fashion to modulate D2R remains to be investigated in the future studies.

Western blot analysis showed a selective reduction of striatal D2R without altering mRNA level of striatal D2R (Fig. 2 and Fig. S1). It is not known how loss of ε-SG specifically affects D2R alone. Amphetamine and its derivatives release dopamine from dopaminergic terminals and cause excessively increased level of extracellular dopamine [32]. Addictive drug is thought to “hijack” normal natural rewards system, such as food or sexual activity, by quickly elevating the level of extracellular dopamine to exert their reinforcing properties. Upon dopamine stimulation, agonist-activated D1R and D2R are rapidly silenced by undergoing endocytosis followed by trafficking to early endosomes. D1R is recycled back to the plasma membrane after desensitization, while D2R is targeted by interaction with GPCR-associated sorting protein and degraded in lysosomes [39]. ε-SG located on the cell membrane may have important roles in regulating endocytic degradation of D2R.

In conclusion, we found significant and selective reduction of striatal D2R in *Sgce* KO mice that is consistent with the neuroimaging results in clinical DYT11 M-D patients [25]. Lack of D2 autoreceptor in dopaminergic neurons may result in enhanced dopamine synthesis in terminals [30] and increased dopamine release after amphetamine injection in the striatum (Fig. 1A). These results support the idea that basal ganglia play very important role in manifesting of dystonia, and suggest that loss of ε-SG may influence the homeostasis of D2R.

### Limitations

The reduction of striatal D2 receptor (Figure 2A) reflects the combination of pre-synaptic and post-synaptic D2 receptors. Although Western blotting result showed the expression level of striatal DAT is not altered and microdialysis experiment showed that extracellular level of dopamine returned to basal level after the stimulation, however the alteration of DAT functions cannot be completely ruled out. There is alternative explanation of obtained results that is due to unknown mechanisms underlying enhanced striatal dopamine release from loss of ε-SG protein, and subsequently increased ligand occupied D2 receptors which became targets of endocytic desensitization process.

## Materials and Methods

### Animals

All experiments were carried out in compliance with the USPHS Guide for Care and Use of Laboratory Animals and approved by the Institutional Animal Care and Use Committee at the University of Alabama at Birmingham with Animal Protocol Number 10008918. *Sgce* heterozygous KO male mice were crossed with C57BL/6 female mice to obtain paternally-inherited *Sgce* heterozygous KO (*Sgce* KO) mice and WT littermates as previously described [9]. Genotyping for *Sgce* KO mice and WT littermates was performed by multiplex PCR using SgceE4U (5′-CTGTAACAACACACTGGAGTAG-3′) and SgceE4D (5′-ACAGCTTTGAACTACTTTGTGCA-3′) primers for the deleted exon 4 locus [9] and DAT-Up (5′-TCCATAGCCAATCTCTCCAGTC-3′) and DAT-Lwt (5′-TTGATGAGGGTGGAGTTGGTCA-3′) primer sets for dopamine transporter gene as an internal control [17]. Mice were housed under a 12-h-light/dark cycle with free access to food and water. All experiments were performed by investigators blind to the genotypes and the animals were decoded after all experiments were finished.

### Drugs administration

Amphetamine (5 mg/kg; Sigma-Aldrich Corporation, St Louis, MO) was dissolved in physiological saline and was injected subcutaneously in a volume of 10 ml/kg, as previously described [40].

### *In vivo* microdialysis

Five-month-old *Sgce* KO mice (n = 6) and their WT littermates (n = 8) were used in microdialysis study, procedure was performed in alert, freely moving mice as previously described [31]. Briefly, mice were anesthetized with ketamine/xylezine (100/10 mg/kg) before the stereotaxic implantation of a probe (Eicom; P-I-6-02) into the striatum at coordinates (+0.0 mm anteroposterior, +2.5 mm mediolateral from the bregma, and −4.4 mm dorsoventral with respect to dura). Probes were secured onto the skull using stainless-steel screws and dental acrylic. Twenty-four hours after surgery, *in vivo* microdialysis was performed on conscious mice. Probe was perfused continuously with artificial CSF (147 mM NaCl, 4 mM KCl, and 2.3 mM CaCl_2_) at a flow rate of 1.5 µl/min. The dialysate was collected in 20-min fractions. Six samples were obtained in order to establish the baseline levels of extracellular dopamine prior to the administration of amphetamine. Dopamine level of microdialysis samples were measured by high-performance liquid chromatography (HPLC) using a reversed phase column (Dionex ESA MD-150×3.2 150 mm Dionex, Chelmsford, MA, USA), as previously described [41]. Probe locations were verified in all mice at the end of the microdialysis experiment. The animal was anaesthetized with ketamine/xylezine (100/10 mg/kg) and perfused transcardially with 4% paraformaldehyde in saline and brains were removed and placed in fixative overnight at 4°C. After equilibrated in 30% sucrose in 0.1 M phosphtate buffer, the brains were sectioned. Coronal sections were cut at 50 µm thickness using a sliding microtome (Histoslide 2000, Reichert-Jung). The sections were Nissl stained.

### Western blot analysis

Striatum was dissected from brains of 5-month-old *Sgce* KO mice (n = 4) and WT littermates (n = 3). The tissue was homogenized in 200 µl of ice-cold lysis buffer contained 50 mM Tris-Cl (pH 7.4), 175 mM NaCl, 5 mM EDTA with a protease inhibitor cocktail tablet (Roche) and followed by sonication for 10 sec. Triton X-100 was added to 1% w/v final concentration and the mixture was incubated on ice for 30 min and centrifuged at 10,000×g for 15 min at 4°C as described earlier [8]. The protein concentration was measured by the Bradford assay with bovine serum albumin (BSA; Fisher Scientific) as standards. The homogenates were mixed with the SDS-PAGE loading buffer and boiled for 5 min, incubated on ice for 1 min, and then centrifuged for 5 min to obtain the supernatant. Of the samples, 30 µg was loaded on a 10% SDS-PAGE and the separated proteins were transferred to the PVDF membrane. After blocking with 5% BSA or 5% non-fat milk (Bio-Rad) TBS-T buffer contained 20 mM Tris-Cl (pH 7.6), 137 mM NaCl, 0.1% Tween 20, the membranes were incubated overnight at 4°C with rabbit anti- dopamine D2 receptor (Millipore, AB5084P) at 1∶500 dilution in 5% BSA TBS-T buffer, goat anti-dopamine D1 receptor (Santa Cruz, sc-31478) at 1∶200 dilution and rabbit anti-dopamine transporter (Millipore, AB1591P) at 1∶500 dilution in 5% non-fat milk TBS-T buffer. Membranes were washed for three times and then incubated with bovine anti-rabbit IgG-HRP (Santa Cruz, sc-2370) or donkey anti-goat IgG-HRP (Santa Cruz, sc-2020) in the 5% non-fat milk TBS-T at room temperature for 1 hour. GAPDH was used as a loading control, and probed with HRP-conjugated GAPDH antibody (Santa Cruz, sc-25778). The band was detected by using Super Signal West Pico Chemiluminescent Substrate (Thermo Scientific). The signals were captured by Alpha Innotech FluorChem FC2 and quantified with UN-SCAN-IT gel (Silk Scientific) software. Each Western blot experiment was repeated three times.

### RNA preparation and RT-PCR analysis

Striatum was dissected from brains of 4-month-old *Sgce* KO mice (n = 2) and WT littermates (n = 2). RNA was extracted from striatum by using RNeasy Fibrous Tissue Mini Kits (Qiagen, Valencia, CA, USA) according to the manufacturer's protocol; cDNA was generated from 1 µg of total RNA using random primers and SuperScript II reverse transcriptase (Invitrogen, Carlsbad, CA, USA). PCR was performed using the cDNA as template with the following primer sets: forward D2S (5′-CACCACTCAAGGATGCTGCCCG-3′), and reverse D2E8 (5′-TTGCTATGTAGACCGTGGTGGGATG-3′) for dopamine receptor D2S; forward D2E7 (5′-GGAGTTTCCCAGTGAACAGGCGG- 3′), and reverse D2E8 for dopamine receptor D2L [42]; forward E1 (5′-CACCCGCGAGCACAGCTTCTTTG-3′), and reverse E2 (5′-AATACAGCCCGGGGAGCATCGTC-3′) for β-actin. PCR products were separated by 2% agarose gel electrophoresis and visualized by ethidium bromide staining [43].

### Statistical analysis

The data are presented as the mean ± standard error of the mean (SEM). The computation was carried out using the STATVIEW 5.0J software (STATVIEW 5.0 J, Tokyo, Japan). The data from the microdialysis were analyzed by repeated ANOVA, comparisons of WT and KO groups were analyzed using student's *t*-test. The data of the Western blot were analyzed by One-way ANOVA. For all analyses, a *p*-value less than 0.05 was considered statistically significant (*p<0.05, **p<0.01).

## Supporting Information

Figure S1 **RT-PCR analysis of striatal D2R.** Semiquantitative RT-PCR analysis of RNA samples extracted from striatum of sgce KO and their WT littermates, β-actin was used as control in this test. The quantified results are shown in the right side. The vertical bars represent means ± SEM of 2 pairs of mice.
(EPS)
